# Time heals all wounds? Naïve theories about the fading of affect associated with autobiographical events

**DOI:** 10.3758/s13421-023-01426-2

**Published:** 2023-04-24

**Authors:** Matthew T. Crawford, Claire Marsh

**Affiliations:** School of Psychology, Victoria University of Wellington, Wellington, New Zealand

**Keywords:** Memory, Autobiographical memory, Affect, Fading affect bias

## Abstract

The current research examined the naïve theories that individuals hold about how affect fades over time. In three studies (with various replications), participants read about positive and negative events and estimated the emotional impact of those events on either themselves or a hypothetical other over different time frames (i.e., 1 week, 1 month, 1 year—Studies 1a–1c) or how long it would take for specific amounts of fade to occur (Studies 2a & 2b). In a final study, participants were directly asked about their beliefs regarding affect fade. Results demonstrated that people have inaccurate expectations about affect fade for positive and negative events. Specifically, participants rate that positive events fade more in the short term, but that negative events fade more in the long term. Results are discussed in terms of how these (incorrect) naïve theories of affect fade relate to metacognitive biases in memory and emotion.

In even the most charmed of lives, negative things are bound to happen. When these events occur, will the negative affect continue unabated, or will it fade over time? What about the affect associated with positive events? Research on affective forecasting shows that while people can accurately predict whether a future event will be positive or negative, they tend to overestimate the intensity and duration of their future emotional reactions (Wilson & Gilbert, 2005). In fact, there is ample evidence of an impact bias and a durability bias in affective forecasts demonstrating that people tend to overestimate both the intensity and the duration of emotional reactions to potential future events—especially negative events (Gilbert et al., 1998). Research on fading affect bias (Walker & Skowronski, 2009; Walker et al., 1997; Walker et al., 2003b) demonstrates that the affect associated with negative autobiographical events fades more quickly (and to a greater degree) than the affect associated with positive events. It has not yet been established in the fading affect bias literature whether people are conscious of this consistent, differential pattern of affect fade despite consistently reporting this pattern of fade for personal event memories. Although fading affect bias and biases in affective forecasting have been treated quite separately, they are both about the duration and strength of affect. In the case of the durability and impact biases, people expect affect to be more durable than it is (i.e., they expect a lack of fade over time) and more intense than it is (i.e., they expect their feelings to be stronger than they are). In the case of fading affect bias, affect fades differentially based on valence (negative fades faster, and to a greater extent, than positive) but it has not yet been established whether people are aware that this occurs, or what beliefs they have about how positive and negative emotions fade over time. Given known differences in what people believe about affect duration and intensity compared with reality (biases in affective forecasting), the current research examines individuals’ naïve theories about how the affect associated with both positive and negative personal event memories changes over time (fading affect bias).

Research across multiple areas of psychology has demonstrated that metacognitive processes (i.e., individuals’ thinking about and understanding of their own cognition) have often direct influences on cognition and behavior (Dunlosky & Metcalf, 2008; Dunlosky et al., 2014; Fleming & Lau, 2014). For example, what people think they already know (i.e., their metacognitive judgment of prior learning) affects how they choose to study (Metcalf & Finn, 2008). The cue-utilization approach (Koriat, 1997) proposes that these subjective judgments of learning are influenced by multiple cues that include the individual’s beliefs about what factors or cues lead to better or worse retention of information. If these beliefs are in error, they are likely to lead to behaviors that produce suboptimal outcomes. Mueller and Dunlosky (2017) demonstrated that when people were led to believe that a particular colour of text was easier to process or was more calming, people estimated that their learning of words in that colour would be better than words presented in a different colour. Importantly, participants’ estimates of their learning were not accurate as there were no differences between recall of words based on colour. The authors suggest that lay beliefs about fluency and memorability or calmness/relaxation and memorability affected estimates even though they were not related to actual performance. Specifically, cues that are believed to lead to greater memorability led participants to overestimate their likely performance on a future memory test. Just as metacognitive beliefs about learning influence estimates of future performance, this paper suggests that beliefs about affect fade influence estimates of the duration of affective states following pleasant and unpleasant life events.

## Impact bias and durability in estimates of future affect

Imagine that your long-term romantic relationship unexpectedly ends: How bad will you feel about it, and for how long will you feel that bad? Questions like these have been used to examine the estimates that people make to life events—and whether these estimates reflect the actual experience of affect over time. Impact bias describes the phenomenon that people tend to overestimate the emotional impact of a future event (Morewedge & Buechel, 2013; Morewedge et al., 2005; Wilson & Gilbert, 2013). This overestimation may be reflected in estimates of affective intensity and/or affective duration. The affective intensity is overestimated regardless of event valence (Buehler & McFarland, 2001), and despite similar previous experiences in which the actual affect experienced was not as extreme as the predictions (Morewedge et al., 2005). Impact bias applies to both hypothetical and actual future events and is thought to occur because atypical (e.g., extreme) instances of an event are more memorable, and as these are brought to mind more easily, they are overweighted in forecasting future affective responses (Kahneman, & Miller, 1986; Morewedge et al., 2005).

Mellers and McGraw (2001) found that people overestimated the strength of their future emotions when predicting grades, the results of pregnancy tests, and whether or not they lost weight while on a diet compared with their actual feelings after these events had occurred. While much of the research on impact bias and affective forecasting is hypothetical or laboratory based, real-world experiments have also demonstrated that people overestimate the impact of an event on their future happiness. Dunn et al. (2003) asked students to rate their future happiness if they were assigned to a college dormitory they considered either desirable or undesirable. These students then rated their actual happiness in the dorms to which they had been randomly assigned for 2 subsequent years. Impact bias was demonstrated in that students in the desirable dorm did not rate themselves as happy as they had estimated, and students in the undesirable dorm were not as unhappy as they had estimated.

In addition to overestimates of affective intensity, cross-sectional surveys based on real events, longitudinal studies, and laboratory-based studies have found that people tend to overestimate the duration of the emotion evoked by both positive and negative events (Gilbert et al., 1998; Wilson et al., 2000). For example, participants who had just voted in the gubernatorial election for their state were asked how happy they were in general, to predict how happy they would be generally in 1-month’s time if their preferred candidate won and if their preferred candidate lost (Wilson et al., 2000). Participants predicted they would be significantly less happy in 1 month if their preferred candidate lost. When asked a month later, those whose preferred candidate won were about as happy as they had expected to be, but those whose preferred candidate lost were not nearly as unhappy as they had estimated they would be. Researchers attribute this inaccuracy to immune neglect—the idea that people are generally unaware of the actions of the “psychological immune system,” which works to ameliorate negative affect outside of awareness. The general lack of insight into the operation of this system means people fail to recognize how effective it is in reducing affective intensity thus, people fail to predict how much (or how quickly) affect fades over time (Wilson & Gilbert, 2005). In essence, the operation of the psychological immune system is not identified as a cue in the production of an estimate of future affect.

## Affect fade

Even though people seem to underestimate the duration of affective states, they do seem to understand that affect does, in fact, fade over time. Finkenauer et al. (2007) compared predicted with actual emotional reactions to passing or failing a driving test. As expected, they found evidence that people (especially those who had failed the test) overestimated the intensity and duration of the emotion elicited by the event. Importantly, even though participants accurately predicted that affect would fade over time, they still vastly underestimated the speed of that fading. Specifically, predicted duration indicated a linear change, whereas actual duration better fit a quadratic pattern. Interestingly, having experienced affect fade for similar events does not result in people making more accurate judgements about future affect retention. For example, Ayton et al. (2007) found that previous experience of the emotional event (i.e., a failed driving test) did not lead to more accurate estimates in affective forecasting. Thus, it seems that our very experiences with affect fade in the past have little bearing on our estimates of affect fade in the future.

As noted above, people do seem to have a belief that affect fades over time (i.e., that it becomes less extreme), but the key to biases in affective forecasting seems to be in terms of either how much or how quickly affect fades. Most of the research has focused on estimating affective responses (i.e., intensity and duration) to negative events. That people overestimate their emotional reactions to future negative events is understandable given the greater impact of negative, as compared with positive, information (Baumeister et al., 2001) in everyday experience. Such negativity biases can be seen in many places. For example, infants privilege negative over positive information when judging social targets (Hamlin et al., 2010; Vaish et al., 2008), and this greater processing and weighting of negative information remains into adulthood (Rozin & Royzman, 2001; Skowronski & Carlston, 1989).

The greater weight paid to negative, compared with positive, leads to a clear prediction—that affect associated with negative events should be retained longer than affect associated with positive events. Wilson et al. (2001) even suggest that the durability bias for positive events reflects the fact that individuals do not remember how short-lived positive emotional reactions tend to be. Beliefs that positive mood is fleeting and that negative emotions endure, however, are counter to the ample evidence demonstrating that affect associated with negative events both fades more quickly and more fully than affect associated with positive events. This differential fading is called fading affect bias (FAB) and has been demonstrated to be a remarkably reliable effect (Landau & Gunter, 2009; Marsh et al., 2019; Ritchie et al., 2015; Walker et al., 1997). Fading affect bias within a daily diary study has been demonstrated to occur in as little as 12 hours post event (Gibbons et al., 2011). That is, even though negative information may attract greater attention, be processed more thoroughly, and recalled with greater likelihood, when it comes to affect fade negative affect does not retain that greater impact.

It seems likely that the fading affect bias occurs outside of any effortful or conscious consideration. Fading affect bias is proposed to serve an emotional resilience function by maintaining more of the positive affect associated with the pleasant events in our lives while simultaneously reducing the negative affect associated with the more unpleasant events (Sedikides & Skowronski, 2020; Walker et al., 2003b; Walker & Skowronski, 2009). Thus, it fits well within Wilson and Gilbert’s (2005) psychological immune system. That is, the differential fading serves to remove affect (especially negative affect) and occurs outside of the awareness of the individual. From a cue-utilization perspective (Koriat, 1997), the cues that these processes are working are not recognized or taken into consideration when forming beliefs about affect fade. As such, estimates reflect “negative is more powerful than positive” beliefs.

Naïve theories about how emotions change over time affect both how individuals remember the past as well as how they make predictions about the future (Ross, 1989). When asked to remember evaluations or feelings from the past, lay theories about the stability of the self over time can introduce bias. For example, people who tend to view the self as stable over time were found to reconstruct earlier evaluations of their romantic relationship in line with their current feelings about the relationship (McFarland & Ross, 1987). Incorrect lay theories have also been indicated in the errors that individuals make regarding affective forecasting. Lowenstein (2007) suggests that one reason for the relatively poor affective forecasting is that people may have incorrect lay theories about how hypothetical events might make them feel were they to happen in real life. He suggests that the durability bias may be driven by the fact that people simply do not have good insight into their unconscious emotion regulation and, as such, do not have appropriate theories about how affect fades over time.

In terms of beliefs about how positive and negative affect fades, discrepancies between estimates and reality could be driven by either erroneous naïve theories about direction or in terms of understanding magnitude differences based on valence. In terms of direction, participants may have different naïve theories about how positive and negative affect changes (or does not change) over time. Igou (2004) proposed that individuals may hold either of two lay theories regarding how emotional intensity is affected by the passage of time. Specifically, it was suggested that these competing lay theories represent either decrease (i.e., fade over time) or continuity (i.e., maintenance/no fade). It could be, for example, that people have a lay theory that negative affect is maintained (i.e., continuity), whereas positive affect decreases over time. Thus, incorrect lay theories about positive and negative fade in terms of decrease or continuity could account for the valence asymmetry.

Alternatively, it is possible that people have the same lay theory about affect fade (i.e., decrease) but are unaware of the magnitude difference that exists for positive and negative affect. That is, if people correctly recognize the direction, but not the correct magnitude of change, then estimates would not reflect the differential demonstrated in fading affect bias. Magnitude errors would emerge, then, not because people expected different directions of fade (i.e., decrease vs. continuity beliefs), but if people were largely unaware of how much or how quickly positive and negative events change over time (and, in particular, that there is a difference between the two). That is, someone may understand that the pain of a break-up will fade with time, but not that it is likely to fade more quickly (and more fully) than the positive affect associated with, for example, starting a new relationship.

## Current research

The current research directly examines the naïve theories that individuals have regarding the fading of affect associated with positive and negative events. The only published study that has examined the influence of estimates about fade versus actual affective fade comes from Ritchie et al. (2009). Using a diary study procedure, participants listed positive and negative events each day. They rated those events in terms of how pleasant or unpleasant they were on that day and how pleasant or unpleasant they thought they would feel about the events 2 weeks later. Participants then rated those same events 2 weeks later. These researchers found that participants’ estimated ratings of emotional change did not fully account for the emergence of the fading affect bias. Specifically, even when controlling for the estimates about how affect would fade (or not) over time, the fading affect bias was still evident.

In the current studies, participants read about positive and negative events that either happened to another person or that they were to imagine happened to themselves. For each scenario, participants estimated the affect that would be felt over different timeframes (Studies 1a–1c), or how long it would take for the affect to fade certain amounts (Study 2). In Study 3, participants were directly asked for their beliefs about the fading of affect associated with positive and negative events. The current research expands on previous research in three ways.

First, even though idiosyncratic nature of the events in the Ritchie et al. (2009) study is a strength in terms of naturalism, it would represent a great amount of variance in terms of event content and affective extremity. By controlling the content and the rated extremity at the time of the event, it is possible to examine beliefs about affect fade in a more controlled manner. The events that were chosen for the scenarios were taken from an extensive database of real events that have been collected in our previous fading affect bias research and represent common positive (e.g., sporting achievement, new job, family trip, new relationship) and negative events (e.g., relationship dissolution, family death, failed test, parents’ divorce).

Second, by having participants estimate their own affect fade over the 2-week period, there is a possibility that this activates not only naïve theories about affect fade itself, but also beliefs about one’s own emotional regulation abilities. Previous research has found that people tend to predict longer durations of affect for others compared with themselves. For example, Igou (2008) found that when asked to imagine a good or a bad grade on a test, students estimated the duration of negative affect after a failure to be shorter for themselves than for another student (with no difference in duration of positive affect following a success). Thus, as people predict the duration of negative affect will be shorter for themselves than others (Igou, 2008), Ritchie et al.’s (2009) finding of fading affect bias could have been enhanced by participant’s beliefs that their own affect has a shorter duration than others.

Finally, Ritchie et al. (2009) had participants estimate their own affect fade at a point 2 weeks in the future. Although research within the fading affect bias literature has demonstrated differential fade within approximately twelve hours of the event, it is important to examine whether peoples’ beliefs about affect fade are different over different timeframes. It seems plausible to expect that people have different expectations about how positive and negative affect is likely to fade over short-, medium-, and long-term frames. To examine this issue, participants rated affect fade at one week, one month, and one year time intervals (Study 1). To examine this in a different manner, in Study 2, participants estimated how long it would take for affect to fade 1 point or 2 points. This way, it will be possible to see whether (and how) beliefs change over different durations.

## Hypotheses

The existing evidence suggests it is likely that people are underestimating the extent to which affect associated with negative events fades compared with the affect associated with positive events. That is, people do expect affect to fade over time, but the magnitude of that expected fade for positive and negative events may not be accurate. With these previous findings in mind, the following predictions were made:

- H1—Direction: It is expected that participants will expect both positive and negative affect to fade over time. This prediction represents a main effect of Time.
- H2—Magnitude: It is expected that that participants will expect greater fade for positive than for negative events over time (i.e., counter to fading affect bias findings). This prediction represents a main effect of Valence.
- H3—Exploratory: No a priori predictions regarding the impact of different timeframes on estimates (i.e., the Time × Valence interaction) were made as no solid theoretical foundation exists for making directional predictions regarding these variables.

## Study 1a

### Method

#### Participants and design

Study 1a participants were 87 students (57 female, 29 male, one nonbinary/other) enrolled in an introductory psychology course (*M*_age_ = 19.1 years, *SD*_age_ = 1.89 years). The studies took the form of a 2 (Target: self vs. other) × 2 (Valence: positive vs. negative) × 3 (Time: 1 week, 1 month, and 1 year) mixed-measures design with repeated factors on the latter two variables. Participants were randomly assigned to either the self (*n* = 43) or other (*n* = 44) rating condition by the randomization function in Qualtrics. Sample size was based on lab experience with previous fading affect bias studies and a basic rule of thumb of 30–40 participants per between-subjects condition. Post hoc analysis of achieved power using G*Power determined that the sample size used demonstrated a power greater than 95% given the Time × Valence effect size and with a .05 alpha. All materials for the studies were presented using Qualtrics online survey software.

#### Materials

Eight vignettes were created based on commonly described events from previous research in which participants were asked to recall and describe positive and negative autobiographical events. There were four positive (sporting achievement, new job, family trip, and new romantic relationship) and four negative (relationship breakup, family death, failed test, and parents’ divorce) events.

An example of a third-person vignette is:

“L.P. is a 19-year-old university student whose romantic partner of 8 months has just unexpectedly broken off their relationship. If asked to rate how pleasant/unpleasant (on a scale from −3 to +3) the event feels at the moment it happened, L.P. would rate it as −3 (*very unpleasant*).”

For the first-person events, the vignette was changed to read:

“Imagine that your romantic partner of 8 months has just unexpectedly broken off the relationship and that if asked to rate how pleasant/unpleasant (on a scale from −3 to +3) the event feels at the moment it happened, you would rate it as −3 (*very unpleasant*).”

For the positive events, two were rated initially as “+3 *very pleasant*” with the other two rated as “+2 *moderately pleasant*.” Similarly, two of the negative events were rated initially as “−3 *very unpleasant*” with the other two rated as “−2 *moderately unpleasant*.” All of the vignettes used in the current studies can be found online (OSF.io https://osf.io/3yu68/). The reported rating “at the time” for both third- and first-party versions was specified in order to have better control over initial intensity as some research has indicated that affective forecasting errors are at least partially due to incorrect estimates of initial intensity (Buehler & McFarland, 2001; Eastwick et al., 2008).

#### Procedure

Participants read each of the eight vignettes in random order. After reading one, participants rated how they thought either the protagonist (other condition) or they themselves (self condition) would feel about the event 1 week, 1 month, and 1 year after it happened. Participants made these judgments on a 7-point scale with endpoints “−3 *very unpleasant*” and “+3 *very pleasant*” (midpoint “0 *neither pleasant nor unpleasant*”). All tasks were self-paced. After evaluating the eight vignettes, participants were debriefed and thanked for their participation.

### Results

Because affect fade for positive and negative events represents movement in different directions (i.e., more positive for negative events and more negative for positive events), the amount of affect fade needed to be put on the same scale. To calculate affect fade for positive valence items, ratings were subtracted from the initial rating. For example, if the initial rating was +3 and the subsequent rating was +2, that would represent 1 point of affect fade. To put the negative valence fade on the same scale, absolute value of the initial rating was taken and added to that of the subsequent rating. Thus, if the initial rating was −3 and the subsequent rating was −2, it would similarly represent 1 point of fade.

**Fig. 1:**
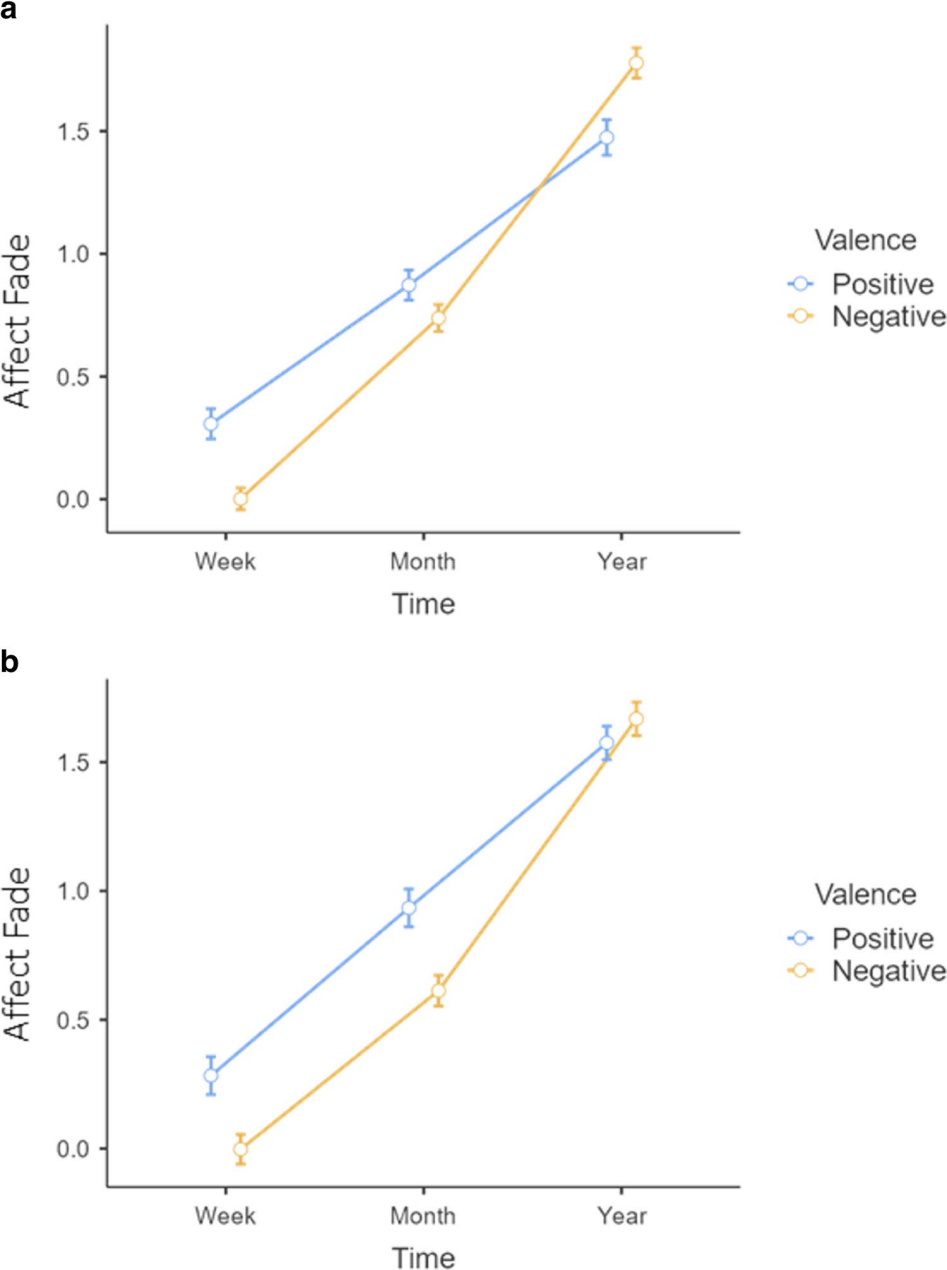
Affect fade as a function of valence and time frame in Study 1a (top chart) and Study 1b (bottom chart). Error bars represent standard error. (Colour figure online)

These measures of affect fade were submitted to a 2 (Target: self vs. other) × 2 (Valence: positive vs. negative) × 3 (Time: week, month, year) mixed-measures analysis of variance (ANOVA), with repeated measures on the latter two variables. The analysis revealed an expected effect of time *F*(2, 172) = 725.30, *p* < .001, η_p_^2^ = 0.89 indicating that people expected affect to fade over time. This is in line with the affect fade hypothesis (H1). The analysis also revealed a significant interaction between Time and Valence, *F*(2, 172) = 35.38, *p* < .001, η_p_^2^ = 0.29. The crossover nature of the interaction can be seen in the top portion of Fig. 1. Simple effects analyses revealed that for the 1-week condition, participants expected the affect associated with positive events to fade more than negative events (*p* < .001, *d* = .48). In the 1-month condition, participants expected marginally greater levels of fading for positive than for negative affect (*p* = .062, *d* = .20). That pattern, however, reverses in the 1-year condition such that participants expected positive events to have faded less than negative events (*p* < .001, *d* = .38). These patterns of results support the magnitude difference hypothesis (H2) for two of the three time-frames—specifically, the shorter-term frames. The nature of the pattern meant that the main effect of valence was not significant (*p* = .47). Additionally, none of the effects including the Target (i.e., self vs. other) manipulation produced a significant effect (smallest *p* = .36). That is, participants demonstrated the same beliefs about affect fade whether they were considering the events from their own perspectives or from the perspective of a third-party observer.

## Study 1b: Replication

### Method

#### Participants and design

Participants in the Study 1b were 91 (62 female, 28 male, one nonbinary/other) students enrolled in an introductory psychology course (*M*_age_ = 19.1 years, *SD*_age_ = 1.52 years). All materials and procedures match exactly those of Study 1a. Participants were randomly assigned to either the self (*n* = 45) or other (*n* = 46) rating condition by the randomization function in Qualtrics. Given the achieved power analysis from Study 1a, the sample size was similar in Study 1b. Post hoc analysis of achieved power using G*Power determined that the sample size used demonstrated a power greater than 95% given the Time × Valence effect size and with a .05 alpha.

### Results

Estimates of affect for positive and negative events at different times from the event were submitted to the same mixed-factors ANOVA as Study 1. Replicating the previous study, the analysis revealed significant Time, *F*(2, 180) = 460.59, *p* < .001, η_p_^2^ = 0.84, and Valence × Time, *F*(2, 180) = 21.07, *p* < .001, η_p_^2^ = 0.19, effects. Follow-up analyses examining valence at each level of time demonstrated that participants expected positive events to fade more than negative events at 1 week (*p* < .001, *d* = .42) and 1 month (*p* < .001, *d* = .50), but not at 1 year (*p* = .23, *d* = .13). The replication study also revealed a significant main effect of Valence, *F*(1, 90) = 3.99, *p* < .05, η_p_^2^ = 0.08, indicating that, overall, participants expected positive events to fade more than negative events (*M*_pos_ = 0.93, *SD*_pos_ = 0.85 vs. *M*_neg_ = 0.76, *SD*_neg_ = 0.90). Again, the Target factor did not result in any significant effects (all *F*s < 1.0).

## Study 1c: Preregistered replication

### Method

#### Power analysis

The sample sizes for the previous studies were based on our own experience with the number of participants that would be sufficient to demonstrate a reliable fading affect bias. Although both Studies 1a and 1b demonstrated good achieved power, for this preregistered replication, the sample size was calculated a priori with G*Power using the average effect size for the Time × Valence interaction (η_p_^2^ = .24), an alpha level set to .05, and power set to 95%. The recommended sample size for a within-subjects *F*-test design with these settings was 60. In order to guarantee that this number was met after expected exclusions, data from 90 individuals was collected.

#### Participants and design

Ninety individuals registered with the online data collection site Prolific.co participated in the current study. Eighteen were excluded (i.e., 20%) due to either failed attention checks (*n* = 6), or evidence of random responding (*n* = 12), leaving a final sample of 72. The average age of the sample was 36.68 years, with 35 identifying their gender as female, 36 as male, and one as nonbinary/other. The study was a 2 (Valence: positive vs. negative) × 3 (Time: week, month, vs. year) fully within-subject design.

#### Procedure

The procedure largely matched that of the previous studies with the following alterations. First, the scale was changed from a 7-point scale (i.e., −3 to +3) to a sliding scale with 60 points. Specifically, the scale used in this study ran from −30 to +30 with every whole-number increment available. This change was made to allow for finer-grained adjustments than was previously the case. Second, the initial ratings of affect were adjusted so that none of them appeared at either end of this new scale. For both the negative and positive events, the events had initial ratings of 18, 20, 22, or 24 (negative events with negative values). This allowed for the possibility of events being rated as more positive or negative than the initial value if participants thought that was likely. Third, only ratings of affective change for others was included as the previous studies showed no differences between expectations for self or other targets. Hypotheses, analysis plans, exclusions, measures, and study design were preregistered (https://osf.io/xf65m).

### Results

Amount of expected change in affect was submitted to a 2 (Valence: positive vs. negative) × 3 (Time: week, month, vs. year) repeated-measures ANOVA. The analysis revealed an expected main effect of Time, *F*(2, 142) = 682.64, *p* < .001, η_p_^2^ = 0.91, indicating that participants expected affect to fade over time. The analysis also revealed a significant main effect of Valence, *F*(1, 71) = 6.37, *p* = .014, η_p_^2^ = 0.08. These effects, however, are subsumed by the Time × Valence interaction, *F*(2, 142) = 182.57, *p* < .001, η_p_^2^ = 0.40. Follow-up analyses examining the Valence effect at each level of Time indicate that participants expected positive events to fade more than negative events after 1 week (*M*_pos_ = 0.73, *SD*_pos_ = 4.01 vs. *M*_neg_ = −0.03, *SD*_neg_ = 3.99; *p* = .013, *d* = .30), no difference at 1 month (*M*_pos_ = 5.24, *SD*_pos_ = 4.53 vs. *M*_neg_ = 5.43, *SD*_neg_ = 4.01; *p* = .66, *d* = .05), and negative events to fade more than positive events at 1 year (*M*_pos_ = 10.50, *SD*_pos_ = 5.36 vs. *M*_neg_ = 14.00, *SD*_neg_ = 4.52; *p* < .001, *d* = .66).

### Combined analysis

The three studies reported here use largely the same methodology (with exceptions as noted for the pre-registered replication) so the data from all three were combined in a single analysis. The analysis revealed, unsurprisingly, a main effect of Time (*F*(2, 494) = 185.75, *p* < .001, η_p_^2^ = 0.43), and a significant interaction between Time and Valence ((*F*(2, 494) = 55.99, *p* < .001, η_p_^2^ = 0.185). Participants expected positive events to fade more in the short-term (i.e., one week, *M*_*pos*_ = 0.42, *SD*_*pos*_ = .22 vs. *M*_*neg*_ = -0.01, *SD*_*neg*_ = 2,17; *p* <.001, *d* = .29) and negative events to fade more in the long-term (i.e., one year, *M*_*pos*_ = 4.11, *SD*_*pos*_ = 5.01 vs. *M*_*neg*_ = 5.27, *SD*_*neg*_ = 6.11; *p* <.001, *d* = .35) with no difference at one month (*M*_*pos*_ = 2.15, *SD*_*pos*_ = 3.16 vs. *M*_*neg*_ = 2.04, *SD*_*neg*_ = 3.08; *p* = .397, *d* = .05). This combined analysis gives us a more reliable estimate of the actual effect size for the findings.

### Discussion

The results of these three studies demonstrate that participants do correctly expect that affect fades over time but that their expectations regarding valence do not match what has been demonstrated via the fading affect bias. That is, participants predicted greater fading of positive compared with negative in the short-term (i.e., a reversal of the fading affect bias pattern), but that over a much longer time period, that negative would fade more than positive (i.e., the normally observed fading affect bias pattern). In terms of continuity versus decline, it seems like, at least for the short term, people expect negative events to remain relatively constant in their intensity. Indeed, across all three studies the expectation of fade for negative events was not significantly different for zero (i.e., no change) at 1-week postevent. As noted earlier, fading affect bias has been demonstrated to happen in as little as twelve hours (Gibbons et al., 2011), so the pattern of predicted affect that our participants estimated for both the one-week and one-month delays is opposite of what has been demonstrated with actual positive and negative autobiographical events. It is only at the 1-year estimate that participants estimate that negative might have faded more than positive affect. It appears, then, that once the event gets far enough in the past to overcome beliefs about the continuity of negative affect, the decline is perceived to be much steeper than for positive affect.

## Study 2a

In Study 1, participants were asked to estimate, for different timeframes, the affect felt for positive and negative events. In Study 2, participants read the same vignettes but were asked to judge how long it would take for the affect associated with the event to fade to certain points. For example, participants reading about an event that the protagonist rated as “−3 *very unpleasant*” were asked to estimate how long (in days) it would take for the person to rate the event as “−2 *moderately unpleasant*” or “−1 *slightly unpleasant*.” This approach was taken in order to provide a different method of assessing beliefs about affect fade. It is clear that people understand that affective intensity decreases over time. Rather than asking for emotional intensity at different timepoints (as in the first series of studies), the current studies (Study 2a & 2b) ask for timeframes to reach certain points of emotional intensity.

### Method

#### Participants and design

Participants in Study 2a were 83 students (60 female, 22 male, one nonbinary/other) enrolled in an introductory psychology course (*M*_age_ = 19.51 years, *SD*_age_ = 3.62 years). The study took the form of a 2 (Target: other vs. self) × 2 (Valence: positive vs. negative) × 2 (Change in Affect: 1 point vs. 2 points) mixed-measures design, with repeated factors on the Valence and Change factors. Sample size was based on the achieved power analyses from the previous studies. All materials were presented via Qualtrics online survey software, and participants completed the study in their own time on their own device. Participants were randomly assigned to either the self (*n* = 41) or other (*n* = 42) rating condition by the randomization function in Qualtrics.

#### Procedure

The vignettes were the same as those used in Study 1. Rather than asking participants how they thought the protagonist would feel at various temporal distances from the events, participants estimated the amount of time (in days) that it would take for the reported affect to fade. Specifically, participants rated how long it would take for either the protagonist (other condition) or themselves (self condition) to change from the “at the time” rating to both 1 point and 2 points less extreme. To use the example from above, participants rated how many days it would take for L.P. to move from “−3 Very Unpleasant” to “−2 *moderately unpleasant*” or “−1 *slightly unpleasant*.” These estimates were made using a slider that ranged from 0 to 600 days (in single-digit increments). Each also included a “longer” option to indicate that it would take more than the 600-day maximum. All tasks were self-paced. After making estimates of time required for affect to fade for all eight vignettes, participants were debriefed and thanked for their participation.

### Results

Estimates of the amount of time required for a particular change were submitted to a 2 (Target: other vs. self) × 2 (Valence: positive vs. negative) × 2 (Change: 1 point vs. 2 points) mixed-measures ANOVA. The analysis revealed an unsurprising main effect of Change, *F*(1, 78) = 35.75, *p* < .001, η_p_^2^ = 0.31*.* As expected, participants rated a 2-point change as taking significantly longer (*M* = 183.77 days, *SD* = 93.16) than a 1-point change (*M* = 129.08 days, *SD* = 98.67) in affect. It is worth noting that both of these estimates represent between 4 and 6 months duration. The analysis also revealed a significant main effect of Target, *F*(1, 78) = 10.71, *p* = .002, η_p_^2^ = 0.12, indicating that participants gave shorter estimates for the self (*M* = 131.86 days, *SD* = 87.92) than participants rating the same events for others (*M* = 181.00 days, *SD* = 103.91). In other words, people expected that it would take less time for affect to fade for themselves, on average, than for others. There was also a nonsignificant interaction between Target and Change, *F*(1, 78) = 3.10, *p* = .08, η_p_^2^ = 0.04. Although not significant there was a trend suggesting that the difference between 1-point and 2-point changes was smaller for first person (*M* = 40.33 days) than for third person (*M* = 74.04 days). Interestingly, Valence had no main or interactive effects (largest *F* = 1.73, *p* = .19). That is, participants did not expect changes in positive and negative events to take different amounts of time.

## Study 2b: Replication

### Method

#### Participants and design

Participants in Study 2b were 88 (60 female, 27 male, one nonbinary/other) students enrolled in an introductory psychology course (*M*_age_ = 19.4, *SD*_age_ = 2.20). All materials and procedures match exactly those of Study 2a. Participants were randomly assigned to either the self (*n* = 44) or other (*n* = 44) rating condition by the randomization function in Qualtrics.

### Results

As in Study 2a, the analysis revealed an expected effect of Change, *F*(1, 84) = 66.63, *p* < .001, η_p_^2^ = 0.44, indicating that a 2-point change (*M* = 191.08 days, *SD* = 116.19) takes longer than a 1-point change (*M* = 129.23 days, *SD* = 90.64). Again, these estimates average out to between about 4–6 months. There was also a significant interaction between Target and Change (*F*(1, 84) = 6.34, *p* = 0.014, η_p_^2^ = 0.07). Follow-up analyses for Target at each level of Change indicated that participants believed that others would demonstrate more rapid fade (collapsing across Valence) of affect than the self when it came to a one-point change (*M*_self_ = 151.13, *SD*_self_ = 106.04 vs. *M*_*oth*_ = 107.33, *SD*_self_ = 75.24; *p* = .036) but no difference in time necessary to produce a two-point change in affect (*M*_self_ = 196.26, *SD*_self_ = 131.60 vs. *M*_oth_ = 185.90, *SD*_oth_ = 100.78; *p* = .97). As in Study 2a, Valence had neither main nor interactive effects in time estimates (smallest *p* = .12).

### Discussion

As expected, participants in Study 2a and 2b demonstrated a belief that affect fades over time and that greater fade requires greater amounts of time. The lack of any main or interactive effects involving valence indicates that participants did not seem to have expectations of differential affect fade for positive and negative events. In fact, there was a tendency to expect positive events to take less time to fade (~130 days) than negative events (~182 days). Even though this effect was not reliable statistically, it is worth noting that the pattern is opposite of what actually occurs (i.e., fading affect bias). Unlike the null findings for the self–other manipulation in Study 1a, the pattern of results in Study 2a did demonstrate a self bias such that participants viewed the amount of time that it would take for affect to fade for themselves was shorter than for others. Interestingly, and somewhat counterintuitively, whether the event was positive or negative had no bearing on this estimate. That participants viewed the fading of negative events to occur more quickly for the self (~137 days) than for others (~187 days) matches the earlier findings of Igou (2008). The fact that they also viewed positive affect fade for the self to take less time (~121 days) than for others (~175 days) is perhaps more surprising.

## Study 3

Thus far, participants’ naive theories about affect fade have been inferred based on the estimates that they provided. A more direct method would be to simply ask people about their beliefs regarding the fading of positive and negative affect. To achieve this, participants in Study 3 were asked whether positive, negative, or neither type of events faded faster and more thoroughly over time.

### Method

#### Participants

Ninety participants were recruited via Prolific.co. The average age of the participants was 37.4 years of age (*SD* = 13.1 years), with 43 identifying as female, 45 identifying as male, and two identifying as nonbinary/other. The sample size was selected in order to ensure at least 80% likelihood of detecting a moderate effect size.

#### Procedure

Participants completed two questions. The first question was “Which fades *faster*? The positive emotions you feel when pleasant things happen, or the negative emotions you feel when unpleasant things happen?” The second question replaced the term “faster” with “more.” Three response options were provided: a) pleasant feelings fade faster [more] than unpleasant feelings; b) unpleasant feelings fade faster [more] than pleasant feelings; c) pleasant and unpleasant feelings fade at the same rate [equally]. The three options were presented in random order for each participant.

### Results

In terms of how quickly positive and negative affect fades, 72.2% (65/90) selected that pleasant feelings fade faster than negative feelings. Only 9 out of 90 (i.e., 10%) believed that negative emotions fade more quickly than positive, with the remaining 17.8% (16/90) believing that positive and negative feelings faded equally fast. A chi-squares test demonstrated that the observed proportions were unlikely to what would be expected by chance, χ^2^(2) = 62.1, *p* < .001. The results for the amount of fading showed a similar pattern, with more believing that positive faded more than negative over time (47/90 or 52.2%) than those believing that negative events faded more than positive events (24/90 or 26.7%) or that they fade equally as much (19/90 or 21.1%). Although weaker than the findings for speed of fade, the Chi-square for amount of fade demonstrated that these proportions were significantly different from what would be expected by chance, χ^2^(2) = 14.9, *p* < .001. Taken together, these results indicate that the majority of people, when directly asked about their beliefs regarding affect fade believe that positive affect associated with pleasant events fades faster and more fully than the negative affect associated with unpleasant events—a pattern of beliefs that is at odds with the actual pattern demonstrated by fading affect bias research.

## General discussion

Taken together, the results of the studies in this paper demonstrate that people have certain beliefs—or naïve theories—about the affect associated with autobiographical events. There is a general expectation that affect fades with time. This belief can be seen with the folk wisdom saying that “Time heals all wounds,” implying that, with time, the pain of negative events will decrease. Although this aphorism refers only to the negative, with experience we learn that all affect fades—both negative and positive—and that seems to carry over into our expectations about affect fade with time.

Even though individuals may recognize that both positive and negative affect fade with time, there seems to be an additional series of beliefs that do not map on to how these events actually fade over time. First, there seems to be a belief that negative affect sticks around longer than positive affect. For example, in Studies 1a–1c, participants expected no fade at all for negative events after a 1-week delay. Additionally, participants demonstrated a pattern in which they expected negative affect to last longer than positive affect. This is perhaps somewhat unsurprising given common statements in the literature suggesting that “bad is stronger than good” (Baumeister et al., 2001, p. 323). Even though negative information often carries more weight (Hamlin et al., 2010; Skowronski & Carlston, 1989), this phenomenon is not equivocal, especially in relation to memory.

One example where good is stronger than bad is the positivity effect which describes a life-span change where a general bias towards negative information shown in youth shifts to a bias towards positivity as age increases (Baltazar et al., 2012; Carstensen & DeLiema, 2018; Kinzler & Shutts, 2008). Positivity biases in memory are not limited to older adults, however, as individuals of all ages strive to elevate the self (D’Argembau & Van der Linden, 2008; Sedikides & Skowronski, 2020). While remaining grounded in reality, people’s memories have many distortions and biases that exist to protect their own self-concept (Conway, 2005; D’Argembau & Van der Linden, 2008; Sedikides & Skowronski, 2020). Positive self-referential information about traits is better remembered than negative information and positive self-relevant events are considered more important and are reactivated more frequently than negative self-relevant events (D’Argembau & Van der Linden, 2008; Rogers, Kuiper & Kirker, 1977).

There was an especially interesting pattern of findings for the different time frames that suggest people have different beliefs about the fading of positive and negative events, depending on the temporal distance from the event itself. For the more short-term frames, participants tended to view positive events as fading more than negative events, or that there was no difference between the amount of fade for positive and negative events. It was only at the longer-term (i.e., 1 year) duration that the patterns started to resemble the fading affect bias pattern of greater fade for negative over positive events. This pattern may represent the fact that people either have different beliefs about affect fade for near versus distant events, or perhaps that their experiences of affect fade inform their estimates depending on temporal distance. This temporal pattern requires additional investigation.

Study 2 shifted from asking about how much fade would occur over specific timeframes to asking how long (in days) it would take for certain levels of fading to occur. This different approach to understanding naïve theories produced the same conclusions. Specifically, people understand that affect fades, but they do not recognize the differential fade of positive and negative events. In fact, participants showed a tendency to expect it to take longer for negative events to fade than for positive events to fade the same amount. It should also be noted that the mean estimates that participants made for how long it would take to shift even one point on the scale—regardless of valence—was significantly longer than what the fading affect literature would indicate (e.g., Gibbons et al., 2011). That is, participants tended to view the time that it would take to decrease 1 point as almost 4 months! This is a clear, albeit indirect, demonstration of expectations about affect fade and time in line with the durability bias discussed by Wilson and Gilbert (2005).

Finally, in Study 3, participants were directly asked for their beliefs about affect fade. The results clearly demonstrated that the majority of respondents expected positive affect to fade both more quickly and more thoroughly than negative affect. In other words, that positive emotions are more fleeting than negative. It should be noted, however, that no particular time frame was indicated for the hypothetical positive and negative events in Study 3. It may be that people were focused more on the immediate aftermath of an event, and because of this close duration were more likely to report this particular pattern of beliefs. Overall, the results of the studies reported in this paper demonstrate that beliefs about affect fade, especially in the short term do not correspond to how affect fades in real life.

### Implications

From a methodological perspective, the current research would indicate that the occurrence of fading affect bias in previous research was not a function of peoples’ naïve theories about affect fade. In most studies, participants are asked to recall past events and rate the pleasantness of that event at the time that it happened and to make the same rating now (i.e., at the time of recollection). This is referred to as the retrospective method for measuring fading affect bias and has been criticized as potentially representing how participants think emotion works. That is, the results may rely on the naïve theories about how affect *should* fade. What the current research indicates is that, if participants are relying on these naïve beliefs, then the pattern of results for the actual experience of affect fade should more closely match the patterns found in the current research. That is, positive should show greater fade over short durations, but negative over longer durations—but this is clearly not the case.

The current work fits broadly into the area of metacognition in that it examines how beliefs about a cognitive process (in this case, how emotions fade) affects subsequent judgements. Our metacognitive beliefs about how cognitive processes work, and the things that are likely to affect those processes, influence the decisions that we make in our daily lives. How many times has a student complained that they did poorly on a test even though they “know the material”? That metacognitive belief may have led the student to stop studying earlier than would have otherwise been required (Metcalf & Finn, 2008). The elderly woman may choose to stay home or choose not to engage with activities because she expects that she will be unable to do so because of her beliefs about age and cognitive ability (Bouazzaoui et al., 2016). The person who believes that aging leads to cognitive decline may treat older family members in ways that take away their sense of agency and control. In terms of the current findings, one may avoid exciting opportunities because of the fear of the emotional impact of failure, specifically, that the pain of that failure will endure more than the potential joy of success.

These errors in beliefs are reminiscent of false beliefs that have been examined in other areas of psychology. Patihis and colleagues (Patihis et al., 2014, 2021) found that students and practicing clinicians are more likely than memory researchers/experts to believe that traumatic events can be automatically repressed and recovered at a much later point in time. From both a legal and a therapeutic perspective, these beliefs have the potential to cause much damage (Otgaar et al., 2019). Similarly, erroneous lay beliefs about the effects of stress on memory can lead to incorrect judgments. For example, Marr et al. (2021) asked different samples of individuals to indicate their agreement with different statements relating to the effects of stress on memory. Although there were some items with high agreement across laypersons and memory experts, there were a number of beliefs that were held by the former but not the latter. Bogaard et al. (2016) demonstrated that both laypersons and experts (i.e., police) held the same false beliefs about verbal and (especially) nonverbal indicators of deception. Members of a jury will be influenced by their perhaps erroneous beliefs that “recovered” memories are real, that stress can only harm recall accuracy, that the police are less susceptible to the effects of stress on memory, or that “shifty eyes” and fidgeting imply deception.

The current work also adds to the small but growing literature examining lay beliefs about emotions. People may have different beliefs about how good or bad, useful, or useless, helpful or harmful emotions are to their lives (Harmon-Jones et al., 2011). Ford and Gross (2019) suggest that beliefs about desirability and controllability of emotions should influence emotion regulation and there is some evidence to support these ideas. It has been shown, for example, that people who feel that emotions are harmful respond more poorly to stressors and have poorer psychological health and well-being (Ford et al., 2017). Similarly, people who believe that emotions are fixed and unchangeable show lower well-being and greater distress than those who believe that one can learn to change emotions (De Castella et al., 2013). The current research did not examine beliefs about emotions themselves, but rather beliefs about how emotions fade over time. Beliefs about fade may be related to beliefs about controllability (e.g., negative emotions are less controllable than positive), but such a conclusion would be speculative and beyond the current work. What is clear is that beliefs about emotion are linked to indicators of well-being.

### Affect fade and well-being

The fading affect bias has been proposed to serve as a general resilience mechanism that helps to keep life looking positive by retaining more positive than negative affect associated with life events (Sedikides & Skowronski, 2020; Walker & Skowronski, 2009; Walker et al., 1997). It seems likely that the fading affect bias occurs outside of any effortful or conscious consideration. Thus, it fits well within Wilson and Gilbert’s (2005) psychological immune system. That is, the differential fading serves to remove affect (especially negative affect) and occurs outside of the awareness of the individual. Evidence of disrupted fading affect bias has been demonstrated by studies showing lower FAB for individuals with higher levels of depression (Marsh et al., 2019; Walker et al., 2003a), alexithymia (Muir et al., 2016), and eating disorder symptomatology (Ritchie et al., 2019). Conversely, grit is positively associated with a stronger fading affect bias (Walker et al., 2019). These findings all indicate that there is a relationship between fading affect bias and other indicators of psychological well-being.

Even though people routinely report, when asked to consider actual events in their lives, that negative affect fades more (and more quickly) than positive affect, this does not seem to impact their estimates of how much affect is likely to fade – either for the self or for a hypothetical other. That people seem to be largely unaware of this difference in fading (and, in fact, seem to expect the opposite), brings up an intriguing question about whether an intervention focusing on this phenomenon might enhance well-being. Specifically, if individuals understood that negative affect fades more (and more quickly) than positive information—the opposite of what they believe is the case—would that have any effect on how they think about current autobiographical events? More to the point, would this recognition then have subsequent effects on other measures of psychological well-being? It seems like a promising question for future research. However, that the fading affect bias largely seems to operate outside of awareness suggests that it is an excellent candidate for the psychological immune system which, as some suggest, works best when left to its own devices (Gilbert et al., 1998; Wilson & Gilbert, 2005).

### Conclusion

Taken together, the current research suggests that individuals hold incorrect beliefs about how affect associated with autobiographical events changes over time. In line with research on the durability bias, participants expect that it will take much longer than what has been demonstrated to be the case for affect (regardless of valence) to fade. The findings also show that peoples’ beliefs about affect fade do not take into account the fact that there are different rates of fading for the affect associated with positive and negative events. Understanding how people think about affect fade, and, in particular, how those beliefs do not match up with reality, likely plays an important role in emotion regulation and emotional well-being.
